# Proliferation of progeria cells is enhanced by lamina-associated polypeptide 2α (LAP2α) through expression of extracellular matrix proteins

**DOI:** 10.1101/gad.263939.115

**Published:** 2015-10-01

**Authors:** Sandra Vidak, Nard Kubben, Thomas Dechat, Roland Foisner

**Affiliations:** 1 Max F. Perutz Laboratories (MFPL), Department of Medical Biochemistry, Medical University of Vienna, Vienna Biocenter (VBC), A-1030 Vienna, Austria;; 2 National Cancer Institute, National Institutes of Health, Bethesda, Maryland 20892, USA

**Keywords:** A-type lamins, nuclear lamina, Hutchinson Gilford progeria, extracellular matrix, cell proliferation regulation, nucleoplasmic lamins, lamina-associated polypeptide

## Abstract

Lamina-associated polypeptide 2α (LAP2α) localizes throughout the nucleoplasm and interacts with the fraction of lamins A/C that is not associated with the peripheral nuclear lamina. The LAP2α–lamin A/C complex negatively affects cell proliferation. Lamins A/C are encoded by *LMNA*, a single heterozygous mutation of which causes Hutchinson-Gilford progeria syndrome (HGPS). This mutation generates the lamin A variant progerin, which we show here leads to loss of LAP2α and nucleoplasmic lamins A/C, impaired proliferation, and down-regulation of extracellular matrix components. Surprisingly, contrary to wild-type cells, ectopic expression of LAP2α in cells expressing progerin restores proliferation and extracellular matrix expression but not the levels of nucleoplasmic lamins A/C. We conclude that, in addition to its cell cycle-inhibiting function with lamins A/C, LAP2α can also regulate extracellular matrix components independently of lamins A/C, which may help explain the proliferation-promoting function of LAP2α in cells expressing progerin.

## Abstract (precis)

A single heterozygous mutation of LMNA generates the lamin A/C variant progerin and causes Hutchinson-Gilford progeria syndrome (HGPS). Vidak et al. show that this mutation leads to loss of LAP2α and nucleoplasmic lamins A/C, impaired proliferation, and down-regulation of extracellular matrix components. Ectopic expression of LAP2α in cells expressing progerin restores proliferation and extracellular matrix expression but not the levels of nucleoplasmic lamins A/C.

Hutchinson-Gilford progeria syndrome (HGPS) is an extremely rare premature aging disease most commonly caused by a heterozygous mutation (c.1824C > T and p.G608G) in exon 11 of *LMNA*, the gene encoding the A-type lamins A and C (De Sandre-Giovannoli et al. 2003; Eriksson et al. 2003). Nuclear lamins are type V intermediate filament proteins and the main components of a filamentous meshwork underlying the inner nuclear membrane (INM), called the nuclear lamina (Dechat et al. 2010a; Gruenbaum and Foisner 2015; Osmanagic-Myers et al. 2015). Lamins provide shape and structural stability to the nucleus but are also involved in many essential cellular processes, such as DNA replication and repair, gene expression, chromatin organization, mechanosensing, and cell proliferation and differentiation. Based on sequence similarities, developmentally regulated gene expression patterns, and biochemical properties, they are classified into A- and B-type lamins. While the major B-type lamins, lamins B1 and B2, are encoded by distinct genes (*LMNB1* and *LMNB2*, respectively), all A-type lamins, of which lamins A and C are the major isoforms, are derived from *LMNA* by alternative splicing. B-type lamins and lamin A are initially expressed as prelamins and undergo multiple steps of post-translational modifications at their C-terminal –CaaX sequence, including farnesylation and carboxymethylation of the cysteine residue (Rusinol and Sinensky 2006). While B-type lamins remain farnesylated and carboxymethylated, prelamin A is further processed by the zinc metalloprotease related to Ste24p (Zmpste24/*FACE1*), which removes the 15 C-terminal amino acids, including the farnesylated and carboxymethylated cysteine residue (Pendas et al. 2002). As a consequence, mature lamin A and lamin C, which never becomes farnesylated since it lacks a –CaaX box, are less lipophilic than B-type lamins and therefore are not only present at the nuclear lamina but also distributed throughout the nucleoplasm as a highly dynamic pool (Moir et al. 2000; Dechat et al. 2010b; Kolb et al. 2011).

The HGPS-causing 1824C > T *LMNA* mutation activates a cryptic splice site, resulting in the expression of a mutant lamin A protein, termed progerin, which harbors a deletion of 50 amino acids within its C terminus, including the Zmpste24 cleavage site (Eriksson et al. 2003). As a consequence, progerin cannot undergo the final proteolytic processing step and retains the C-terminal farnesyl group, leading to its stable association with the INM (Dechat et al. 2007). Progerin acts in a dominant-negative fashion and induces various cellular defects—including alterations in nuclear shape and structure, mechanotransduction, gene expression, various signaling pathways, DNA repair, and chromatin organization—and subsequently leads to premature senescence (Ghosh and Zhou 2014; Gordon et al. 2014).

Previous studies reported lamina-associated polypeptide 2α (LAP2α) down-regulation as one of the characteristics of the HGPS cellular phenotype (Scaffidi and Misteli 2006; Cenni et al. 2011). LAP2α is the largest of six LAP2 isoforms expressed in mammals (Gesson et al. 2014). In contrast to most other LAP2 isoforms, which are integral proteins of the INM, LAP2α lacks a transmembrane domain and localizes throughout the nuclear interior (Dechat et al. 1998, 2004), where it interacts with chromatin (Vlcek et al. 1999; Zhang et al. 2013). Furthermore, LAP2α specifically binds to A-type lamins in interphase cells and has been implicated in the regulation and stabilization of the nucleoplasmic pool of A-type lamins (Dechat et al. 2000; Naetar et al. 2008). A-type lamins and LAP2α have been shown to directly interact with retinoblastoma protein (pRb) (Markiewicz et al. 2002; Dorner et al. 2006), a prominent regulator of the cell cycle. As this interaction is important for the localization, anchorage, and stability of pRb within the nucleus and regulates pRb-dependent repression of E2F target genes, nucleoplasmic lamin A/C–LAP2α is implicated in cell cycle regulation (Gesson et al. 2014). Previous studies have shown that loss of LAP2α leads to hyperproliferation of tissue progenitor cells in LAP2α-deficient mice and impaired cell cycle arrest during contact inhibition in cell culture (Pekovic et al. 2007; Naetar et al. 2008). In contrast to LAP2α deficiency, LAP2α overexpression leads to a decrease in the proliferation rate and a reduction in E2F transcription activity (Dorner et al. 2006).

As it has been suggested that nucleoplasmic A-type lamins together with LAP2α have an important role in the regulation of cell proliferation (Gesson et al. 2014), which has been found impaired in progerin-expressing cells (Bridger and Kill 2004; Hernandez et al. 2010), we set out to determine the role of LAP2α in the progression of the cellular HGPS phenotype. Here we demonstrate in primary HGPS patient fibroblasts and human telomerase reverse transcriptase (hTERT) immortalized fibroblasts that progerin expression down-regulates LAP2α expression at the transcriptional and translational level, causes loss of nucleoplasmic lamin A/C, and leads to impaired cell proliferation. The loss of LAP2α is not a consequence of progerin-induced cell cycle exit or senescence but rather causes the proliferative defects of HGPS fibroblasts because reintroduction of LAP2α into progerin-expressing cells rescues proliferation. Re-expression of LAP2α in progerin-expressing cells does not rescue the nucleoplasmic pool of A-type lamins but increases expression of several extracellular matrix (ECM) proteins. In addition, cultivation of progerin-expressing cells on a preformed ECM derived from GFP-progerin cells re-expressing LAP2α promotes their proliferation. Our data suggest that LAP2α may rescue proliferation of progerin-expressing cells by modulating the ECM expression independently of the nucleoplasmic LAP2α–lamin A/C complex.

## Results

### LAP2α is down-regulated in HGPS patient fibroblasts depending on progerin expression levels

**Figure 1.**
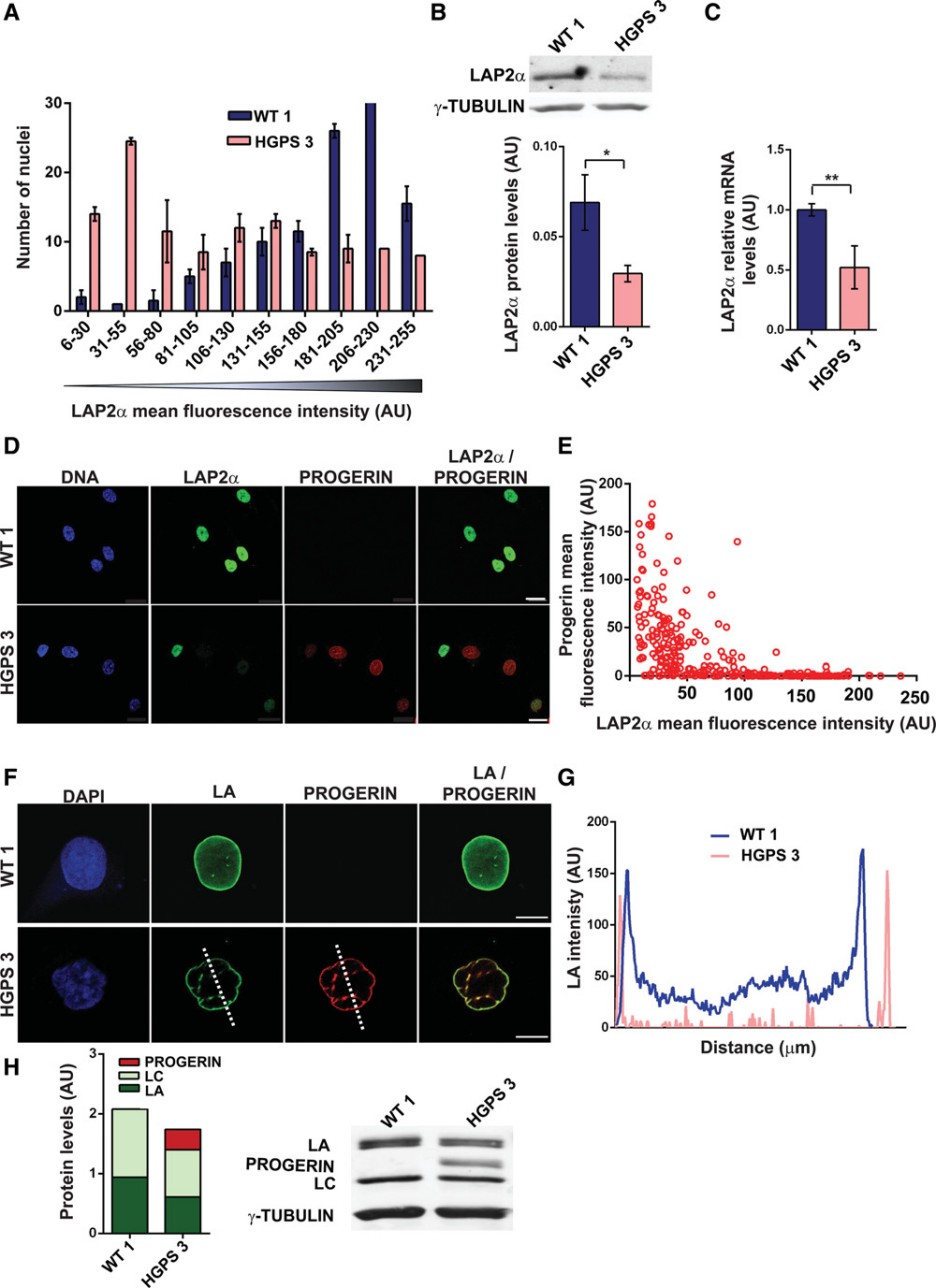
LAP2α down-regulation and decrease in nucleoplasmic A-type lamins correlate with high progerin expression levels in HGPS fibroblasts. (*A*) Mean LAP2α fluorescence intensities of 115 nuclei in wild-type (WT) and HGPS fibroblasts were plotted in a histogram. (*B*) Quantitative immunoblot analysis of LAP2α protein in total cell lysates. *n* = 3; *P* = 0.01. (*C*) LAP2α mRNA levels relative to β-actin mRNA determined by qRT–PCR. *n* = 3; *P* = 0.001. (*D*) HGPS and wild-type fibroblasts were analyzed by immunofluorescence microscopy using anti-LAP2α (green) and anti-progerin (red) antibodies and DAPI (DNA; blue). Bar, 20 µm. (*E*) Mean progerin fluorescence intensities were measured in HGPS fibroblasts and plotted over mean LAP2α intensities. *n* = 115. (*F*) HGPS or wild-type fibroblasts were processed for immunofluorescence microscopy using anti-lamin A/C (LA/C; green) and anti-progerin (red; does not react with LA/C) antibodies. DNA was stained with DAPI (blue). Bar, 10 µm. (*G*) The mean fluorescence intensity of the LA/C signal was measured across nuclei (dotted line) and plotted. (*H*) Quantitative immunoblot analyses of HGPS and wild-type fibroblast in total cell lysates using anti-LA/C and anti-γ-tubulin antibodies. *n* = 3; *P* = 0.15.

Previous studies have shown that total LAP2 as well as LAP2α levels are decreased in HGPS cells (Scaffidi and Misteli 2005, 2008; Cenni et al. 2011; Zhang et al. 2011), but it remained unclear whether this is causally linked to the progression of the cellular HGPS phenotype. To investigate the down-regulation of LAP2α in more detail, we analyzed mid-passage (p10–p13), passage-matched dermal fibroblasts derived from HGPS patients or healthy control individuals by immunofluorescence microscopy. We used three different HGPS cell lines: HGADFN003 (2 yr, shown as HGPS 1), HGADFN155 (1 yr, shown as HGPS 2), and AG11513 (12 yr, shown as HGPS 3). As all control cells behaved similarly, HGMDFN168 (WT 1) is shown as the control. While the LAP2α-specific signal was high in most nuclei of control fibroblasts, LAP2α signal intensities were clearly reduced in the nuclei of HGPS fibroblasts (Fig. 1A,D; Supplemental Fig. S1A). Quantification of the average mean fluorescence intensity of LAPα in nuclei from HGPS fibroblasts (*n* = 300) revealed an overall reduction in LAP2α levels by 15%–50% (depending on the severity of the HGPS phenotype of the respective fibroblast lines) compared with control cells (Supplemental Fig. S1B). The decrease in LAP2α protein levels in HGPS cells was confirmed by immunoblotting (Fig. 1B). Furthermore, quantitative RT–PCR (qRT–PCR) analysis revealed a reduction of LAP2α mRNA levels, suggesting that, in HGPS fibroblasts, LAP2α down-regulation occurs also at the transcriptional level (Fig. 1C).

As progerin expression levels are highly heterogeneous in individual cells of HGPS cell cultures (McClintock et al. 2006), we investigated whether loss of LAP2α is related to progerin levels using double-immunofluorescence microscopy of mid-passage HGPS fibroblasts. We found a clear correlation of LAP2α down-regulation with progerin expression levels. HGPS fibroblasts expressing high levels of progerin had greatly reduced LAP2α levels as compared with low-progerin-expressing HGPS fibroblasts and progerin-lacking control fibroblasts (Fig. 1D). Quantification of the progerin and LAP2α signals in nuclei of HGPS fibroblasts (*n* = 285) revealed that high progerin signals correlated with low LAP2α signals, while nuclei with high LAP2α levels had nearly no progerin expression (Fig. 1E). To investigate whether LAP2α down-regulation is a common progeria-related phenotype, we examined LAP2α and progerin levels in two other patient cell lines derived from younger patients (HGPS 1 and HGPS 2) in addition to the one analyzed before, derived from an older patient (HGPS 3), at mid-passage (p11–p13) by double immunofluorescence. Average mean fluorescence intensities of LAP2α and progerin (250 nuclei) in these HGPS cell lines revealed that the extent of LAP2α down-regulation correlated with progerin expression levels and patient age (Supplemental Fig. S1B). Previous findings suggested that progerin levels and severity of cellular HGPS phenotypes observed in cultured HGPS fibroblasts also increased with passage numbers (McClintock et al. 2006; Cao et al. 2011). In line with these studies, we found that the average mean LAP2α fluorescence intensity in HGPS fibroblasts (HGPS 2) decreased during passage, correlating with increased progerin levels (250 nuclei measured at 10 vs. 20 passages) (Supplemental Fig. S1C).

### The nucleoplasmic pool of A-type lamins is decreased in HGPS fibroblasts

Down-regulation of LAP2α has previously been reported to lead to a reduction of the nucleoplasmic pool of A-type lamins (Pekovic et al. 2007; Naetar et al. 2008; Naetar and Foisner 2009). We therefore tested by double-immunofluorescence microscopy whether HGPS fibroblasts expressing high levels of progerin—and thus low levels of LAP2α—display reduced nucleoplasmic A-type lamin levels compared with control cells using anti-lamin A/C antibody (also detecting progerin) and anti-lamin A antibody that does not recognize lamin C and progerin (Dechat et al. 2007). With both antibodies, the lamin-specific signal was detectable at the nuclear periphery (corresponding to the nuclear lamina) and throughout the nuclear interior (corresponding to nucleoplasmic lamins) in control fibroblasts, while it was mostly restricted to the nuclear rim in HGPS fibroblasts (Fig. 1F,G; Supplemental Fig. S1D,E). The loss of nucleoplasmic lamins in HGPS cells is not caused by a reduced expression of A-type lamins, as immunoblot analysis of control and HGPS fibroblast lysates revealed no significant changes in total levels of A-type lamins (lamin A, lamin C, and progerin) (Fig. 1H; Dahl et al. 2006).

### Loss of LAP2α precedes progerin-dependent proliferation defects in HGPS cells

**Figure 2.**
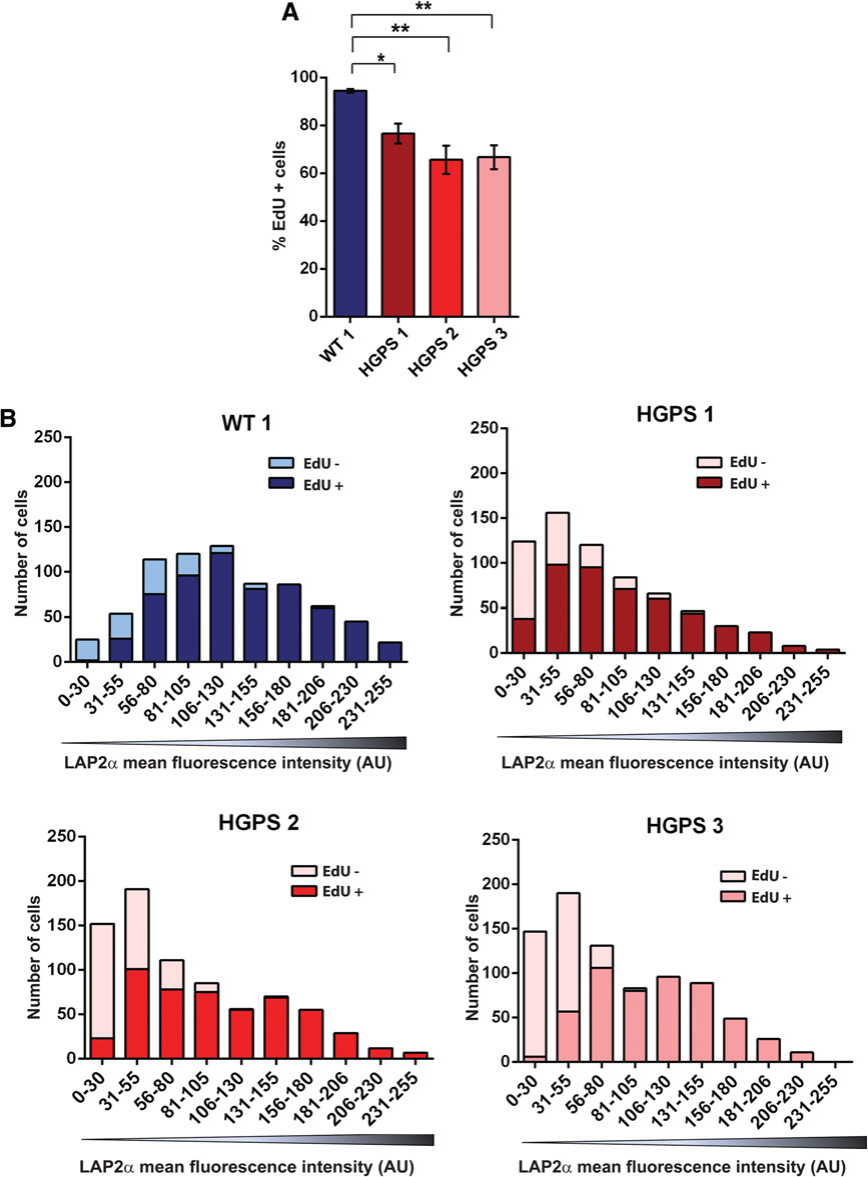
Loss of LAP2α correlates with impaired proliferation. (*A*) Wild-type (WT) and three different HGPS (HGPS 1, HGPS 2, and HGPS 3) fibroblast cultures were grown in medium containing EdU. The percentage of cells showing EdU incorporation (% EdU^+^ cells) was determined after 40 h. HGPS 1, *P* = 0.015; HGPS 2, *P* = 0.0013; HGPS 3, *P* = 0.0009; *n* = 500. (*B*) Mean fluorescence LAP2α intensity of 600–700 nuclei from one control cell line and HGPS 1, HGPS 2, and HGPS 3 cell lines was measured and plotted against the EdU content in each cell.

Cell cycle exit and premature senescence are hallmarks of HGPS cells in culture (Bridger and Kill 2004; McClintock et al. 2006; Espada et al. 2008). Transient (quiescence) or stable (terminal differentiation and senescence) cell cycle exit has also been found to correlate with down-regulation of LAP2α expression and loss of nucleoplasmic lamins (Markiewicz et al. 2002, 2005; Naetar et al. 2007, 2008). We therefore tested whether the observed loss of LAP2α and nucleoplasmic lamins in HGPS cells correlated with progerin-induced proliferation defects. Forty-hour EdU incorporation assays in mid-passage cultures (p11–p13) of all three HGPS cell lines used in this study revealed a reduced number of EdU-positive proliferating cells compared with the healthy control (60%–70% vs. >90%; *n* = 3; *P* < 0.05) (Fig. 2A). To analyze a potential link between proliferation and LAP2α expression, we performed EdU incorporation followed by immunofluorescence microscopy using anti-LAP2α antibody. Measuring the mean LAP2α fluorescence intensity in 600–700 nuclei revealed that EdU-negative, nonproliferating cells contained low LAP2α levels, while EdU-positive cells always had high LAP2α levels (Fig. 2B).

**Figure 3.**
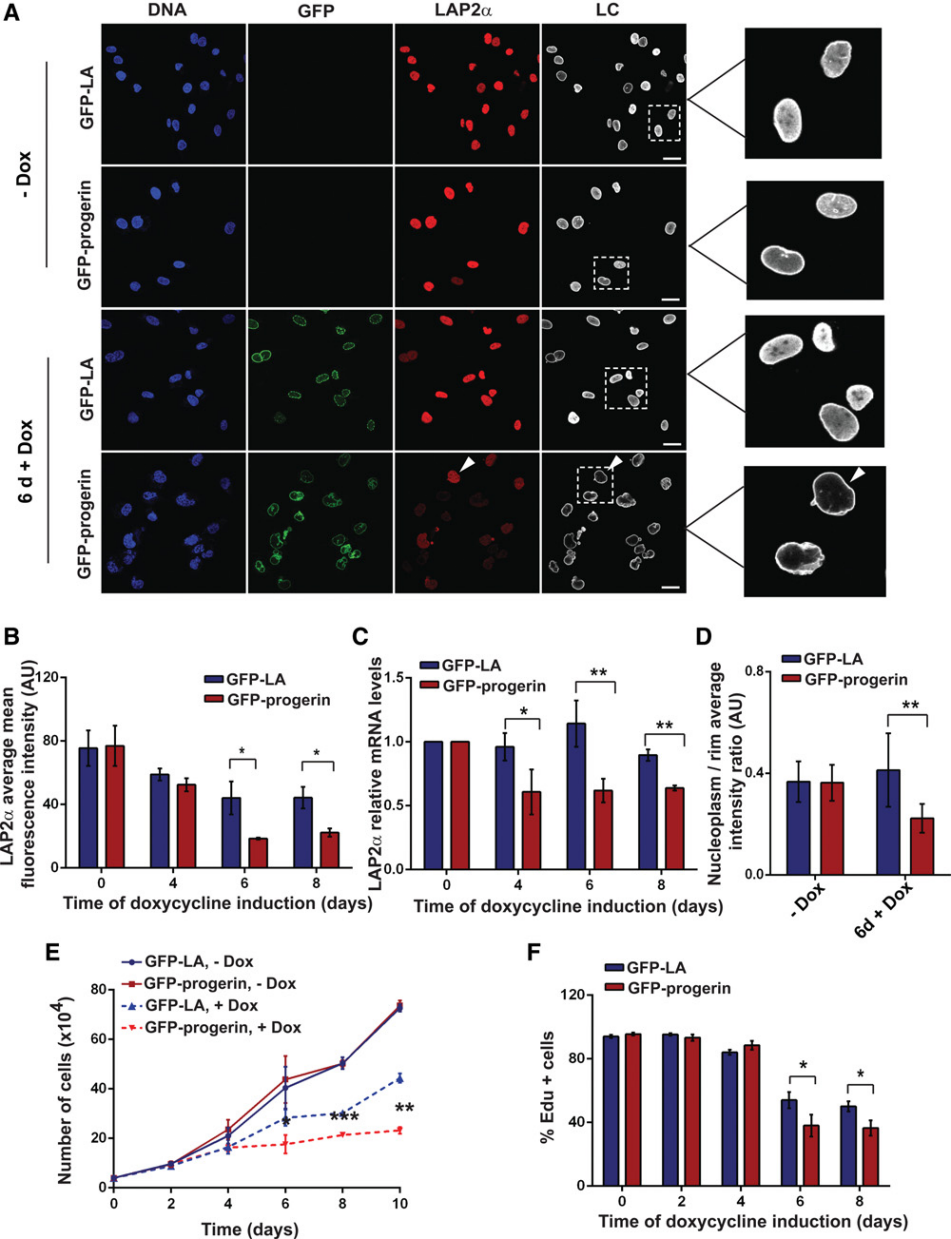
Ectopic expression of GFP-progerin causes LAP2α down-regulation and cell cycle exit in hTERT immortalized skin fibroblasts. (*A*) Immunofluorescence microscopic analysis of hTERT immortalized Tet-on skin fibroblasts inducibly expressing GFP-lamin A or GFP-progerin in their uninduced state (−Dox) and 6 d after induction with doxycycline (+Dox) using anti-LAP2α and anti-lamin C (LC; does not recognize LA or progerin) antibodies. DNA was stained with DAPI. Arrowheads show nucleus containing LAP2α signal. Bar, 20 µm. (*B*) GFP-lamin A and GFP-progerin cells were processed for immunofluorescence using anti-LAP2α antibody prior doxycycline induction (0) and 4 d, 6 d, and 8 d after induction. The average mean LAP2α fluorescence intensity was plotted in a histogram. *n* = 200; *P* = 0.047 for 6 d; *P* = 0.024 for 8 d. (*C*) LAP2α mRNA levels relative to β-actin mRNA were determined by qRT–PCR prior to doxycycline induction and 4 d, 6 d, and 8 d after induction and were normalized to the respective uninduced state. *n* = 3; *P* = 0.007 for 4 d; *P* = 0.001 for 6 d; *P* = 0.003 for 8 d. (*D*) Ratios of nucleoplasmic to peripheral mean lamin C fluorescence intensities were calculated from 20 GFP-lamin A and GFP-progerin nuclei in immunofluorescence images and plotted in a histogram. *P* = 0.000014. (*E*) Growth curves of hTERT immortalized fibroblasts containing GFP-lamin A (blue) or GFP-progerin (red) grown for 10 d in medium with (+Dox) or without (−Dox) doxycycline. *n* = 4. (*F*) GFP-lamin A and GFP-progerin hTERT immortalized fibroblast were grown in medium containing EdU. The percentage of cells showing EdU incorporation (% EdU^+^ cells) was determined after 40 h at the indicated post-induction time points. *n* = 250; *P* = 0.033 for 6 d; *P* = 0.0057 for 8 d.

These data show that the progerin expression-linked loss of LAP2α in HGPS cells is correlated with proliferation defects. However, they do not show whether loss of LAP2α is simply a consequence of progerin-caused cell cycle exit or plays an active role in promoting cell cycle exit. To address this question, we analyzed LAP2α down-regulation in relation to progerin expression in a tightly controllable system. We generated hTERT immortalized skin fibroblast cell lines containing doxycycline-inducible GFP-lamin A or GFP-progerin constructs. Ectopic proteins were detectable within 1 d following doxycycline addition and plateaued at 6 d (Supplemental Fig. S2A–C). Doxycycline did not affect proliferation of parental cells transfected with empty vector (Supplemental Fig. S2D). Both GFP-lamin A and GFP-progerin accumulated at the nuclear periphery, and hTERT fibroblasts expressing GFP-progerin frequently showed lobulated and misshapen nuclei (Supplemental Fig. S2A). Similar to the HGPS patient cells, LAP2α levels were decreased in hTERT fibroblasts expressing GFP-progerin compared with uninduced or GFP-lamin A-expressing cells (Fig. 3A). Quantitative immunofluorescence microscopy revealed that LAP2α protein levels started to decline 5–6 d after doxycycline induction and reached a minimum 7–8 d after induction (Fig. 3B). A decrease in LAP2α mRNA levels were detectable already 4 d after doxycycline addition (Fig. 3C), when LAP2α protein levels were still unchanged (Fig. 3B). Treatment of cells with proteasomal inhibitor MG132 for up to 24 h did not reveal differences in LAP2α protein stability between wild-type and HGPS fibroblasts (Supplemental Fig. S3A). In addition, immunoblot analysis of wild-type and LMNA^−/−^ mouse embryonic fibroblasts revealed identical LAP2α levels, showing that lack of lamins A/C does not lead to faster degradation of LAP2α (Supplemental Fig. S3B). These data suggest that progerin expression initially down-regulates LAP2α mRNA levels rather than affecting LAP2α protein turnover. Furthermore, immunofluorescence analysis of primary fibroblasts and progerin-expressing hTERT fibroblasts revealed decreased E2F-1 protein levels, correlating with loss of LAP2α (Supplemental Fig. S3C), and mRNA levels of E2F target genes were reduced in progerin cells (Supplemental Fig. S3D). As LAP2α is an E2F target gene (Parise et al. 2006), lower levels of E2F may account for decreased LAP2α mRNA levels.

In addition, unlike GFP-lamin A, induction of GFP-progerin caused loss of nucleoplasmic A-type lamins, as shown by immunofluorescence microscopy using a lamin C-specific antibody (Fig. 3A). The ratio of nucleoplasmic over peripheral mean lamin C intensities was decreased by 50% in GFP-progerin-expressing versus GFP-lamin A-expressing cells at post-induction day 6 (*n* = 20) (Fig. 3D). Interestingly, a decrease of nucleoplasmic A-type lamins was detectable also in GFP-progerin-expressing hTERT fibroblasts that still contained normal LAP2α levels (Fig. 3A, arrowheads), suggesting that the loss of nucleoplasmic lamins A/C was caused by progerin expression rather than LAP2α depletion.

Having analyzed the kinetics of LAP2α down-regulation upon progerin expression, we next investigated proliferation of cells following doxycycline induction. While uninduced GFP-lamin A and GFP-progerin hTERT fibroblasts exhibited similar growth rates when monitored for 10 d, both GFP-lamin A-expressing and GFP-progerin-expressing cells showed a reduced growth rate compared with uninduced cells after 4 d of doxycycline treatment (Fig. 3E). However, at days 5–6 after induction, when LAP2α mRNA levels were already low and LAP2α protein levels started to decrease, the GFP-progerin-expressing fibroblasts dramatically slowed down and subsequently stopped proliferating, while GFP-lamin A-expressing cells continued to grow (Fig. 3E). The proliferation defect of the GFP-progerin-expressing fibroblast cultures at day 6 after induction were also detectable in 40-h EdU incorporation assays (Fig. 3F). These data suggest that the loss of LAP2α in progerin-expressing cells precedes detectable proliferation defects and therefore is not likely a consequence of impaired cell proliferation but may contribute to the growth defects.

### Re-expression of LAP2α rescues the proliferation of GFP-progerin cells but does not rescue the nucleoplasmic pool of A-type lamins

**Figure 4.**
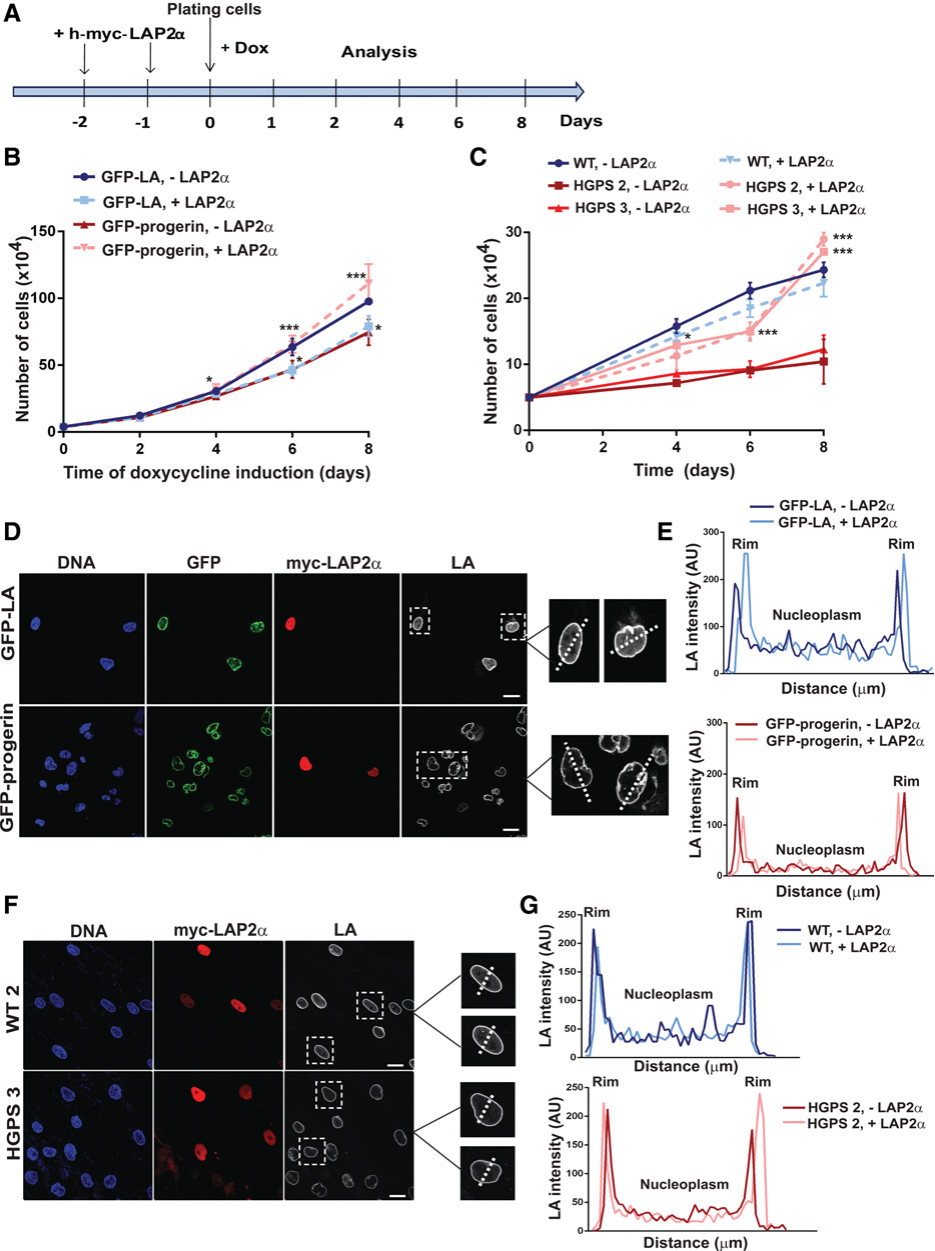
LAP2α enhances proliferation of progeria cells independently of nucleoplasmic lamins. (*A*,*B*) hTERT cells were transfected with either a control GFP-expressing (pHR-GFP) or a h-myc-LAP2α-expressing construct on two consecutive days prior to the induction of lamin A or progerin expression and counted every other day for 8 d after induction. *n* = 4. (*C*) Primary human fibroblasts were transfected in the same way as in *A* and counted every other day for 8 d. *n* = 3. (*D*,*F*) Immunofluorescence analysis of h-myc-LAP2α transfected cells using anti-myc (red) and lamin A-specific (LA; grayscale) antibodies and DAPI (DNA; blue) Bar, 20 µm. (*E*,*G*) Mean fluorescence intensities of the lamin A (LA) signal were measured across nuclei (dotted line) and plotted.

Since down-regulation of LAP2α clearly preceded the decrease in cell proliferation in the GFP-progerin-expressing cells, we investigted whether overexpression of LAP2α in the hTERT fibroblasts can rescue the impaired cell growth. Therefore, we ectopically expressed myc-tagged human LAP2α using a lentiviral transduction system, with which we achieved transfection rates of up to 90% and expression levels of myc-tagged human LAP2α comparable with that of endogenous LAP2α (Supplemental Fig. S4A,B). For cell growth analyses, cells were transfected with the LAP2α-encoding or a GFP-encoding control construct on two consecutive days prior to the induction of GFP-lamin A or GFP-progerin and analyzed up to 8 d after induction (Fig. 4A). Fibroblasts transfected with a GFP control plasmid began to slow down proliferation between days 5 and 6 after GFP-progerin induction compared with GFP-lamin A induction (Fig. 4B, blue and red full lines). Expression of myc-LAP2α in GFP-lamin A-expressing cells (Fig. 4B, blue dashed line) and uninduced control cells (Supplemental Fig. 4C) also resulted in a slow down of proliferation. This is in agreement with previous studies showing that overexpression of LAP2α leads to impaired cell growth (Dorner et al. 2006; Naetar et al. 2008). Strikingly, however, ectopic expression of LAP2α in the GFP-progerin-expressing hTERT fibroblasts partially rescued the growth inhibitory effect of GFP-progerin to the levels of GFP-lamin A-expressing cells (Fig. 4B, red dotted line).

To demonstrate that the proliferation-promoting effect of LAP2α in progerin-expressing hTERT cells is independent of telomerase expression, we introduced human myc-tagged LAP2α into primary HGPS fibroblasts (HGPS 2, p19; and HGPS 3, p21) and one wild-type control (GM02037, WT 2, p20, age matched to HGPS 3). In both HGPS cell lines but not the control, we observed a rescue of proliferation upon reintroduction of LAP2α (Fig. 4C).

Next, we investigated how LAP2α expression can rescue cell proliferation in progerin-expressing cells but slows down proliferation in GFP-lamin A-expressing and uninduced control cells. Our previous data suggested that both nucleoplasmic A-type lamins and LAP2α cooperatively regulate cell proliferation (Naetar et al. 2008; Naetar and Foisner 2009; Pilat et al. 2013). As shown before (Fig. 1F; Supplemental Fig 4D,F), expression of progerin led to a reduction in the nucleoplasmic pool of A-type lamins. Interestingly, ectopic expression of myc-LAP2α did not rescue the nucleoplasmic pool of lamins A/C in both hTERT progerin-expressing cells (Fig. 4D,E) and primary HGPS fibroblasts (Fig. 4F,G). These findings may explain the different effects of LAP2α expression on cell proliferation in control versus progerin-expressing cells. While the increase in LAP2α in cells containing nucleoplasmic A-type lamins reduces proliferation as shown previously (Dorner et al. 2006; Pilat et al. 2013), it had no inhibitory effect on cell growth in progerin-expressing cells that lack nucleoplasmic lamins A/C. We therefore concluded that LAP2α in progerin cells may have a nucleoplasmic lamin A/C-independent function that promotes proliferation.

### LAP2α enhances proliferation of progerin cells by up-regulation of ECM components

In an attempt to identifiy potential lamin A-independent growth-promoting functions of LAP2α in progeria cells, we tested the expression of ECM proteins in these cell systems, as our previous genome-wide expression-profiling analyses in LAP2α-deficient mouse myoblasts revealed a down-regulation of ECM proteins compared with wild-type myoblasts (Gotic et al. 2010). Furthermore, defects in ECM protein production have been causally linked to proliferation defects in several HGPS models (Csoka et al. 2004; Hernandez et al. 2010; de la Rosa et al. 2013). Proliferative defects of adult mouse fibroblasts harboring a HGPS-linked *Lmna* mutation (Δ9*Lmna*) were rescued upon growth on ECM derived from wild-type cells (Hernandez et al. 2010), and prelamin A-expressing ZMPSTE24-deficient mouse cells proliferated normally in a mosaic mouse model containing wild-type and ZMPSTE24-deficient cells in its tissues (de la Rosa et al. 2013).

We analyzed mRNA levels of various ECM proteins in primary human HGPS versus control fibroblasts and in GFP-progerin-expressing versus GFP-lamin A-expressing hTERT fibroblasts by qRT–PCR (Supplemental Fig. 5A,B). We focused on ECM components previously reported to be down-regulated in mouse models and patient cells: *Aspn*, *Col12A1*, *Col11A1* (Hernandez et al. 2010), *Col12A1*, *Col1A1*, *Timp2* (Gotic et al. 2010), *MMP15* (Csoka et al. 2004), and *Col3A1*, frequently found in association with type I collagen. In the progeria cell line HGPS 3 that expresses low LAP2α and high progerin levels (Fig. 1; Supplemental Fig. S1), ECM mRNA levels were significantly reduced (Supplemental Fig. S5A), while, in cell line HGPS 1 that expresses low progerin and normal LAP2α levels (Supplemental Fig. S1), ECM expression was similar to that of wild-type cells. In line with this, GFP-progerin-expressing hTERT fibroblasts showed reduced levels of ECM mRNAs compared with respective uninduced controls (Supplemental Fig. S5B). Also, GFP-lamin A expression caused down-regulation of some ECM mRNAs, although to a lesser extent than GFP-progerin (Supplemental Fig. S5B).

**Figure 5.**
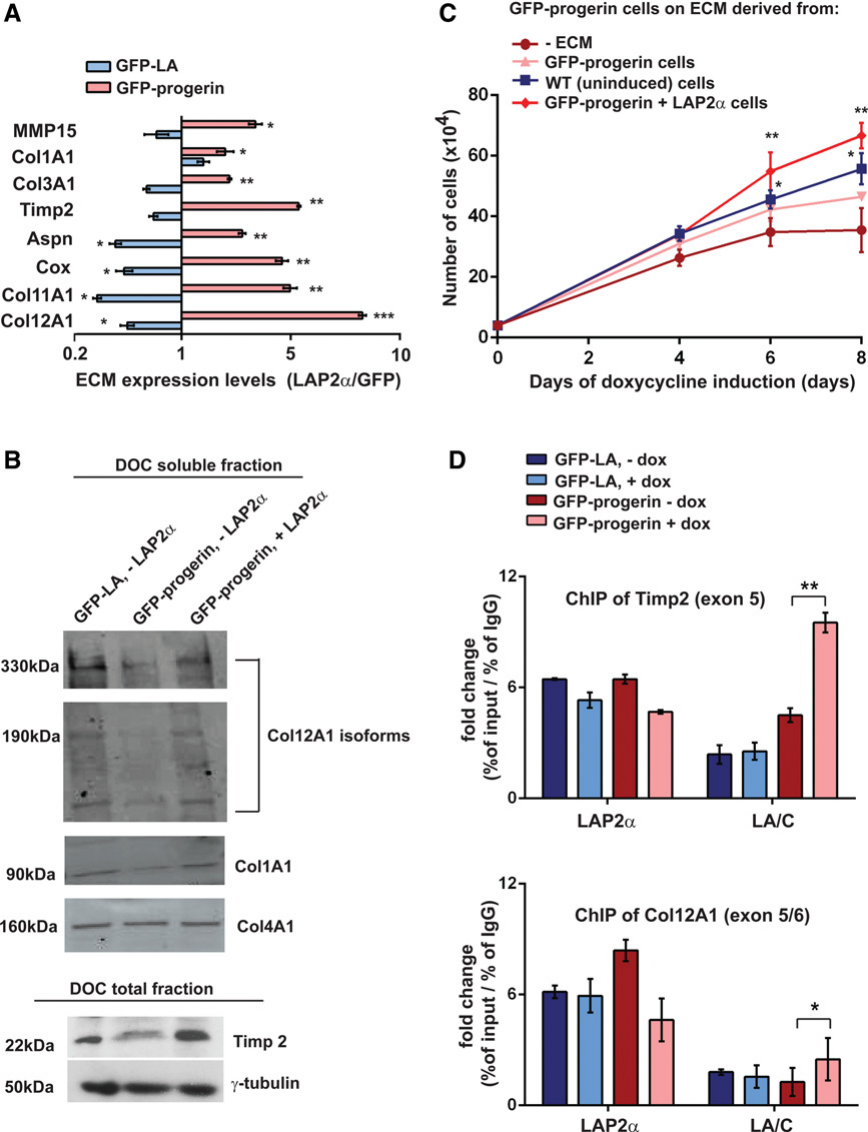
LAP2α enhances proliferation by affecting the expression of ECM proteins. (*A*) qRT–PCR analysis of ECM mRNA levels in h-myc-LAP2α-expressing cells at day 4 after induction relative to β-actin and normalized to the respective control GFP-expressing cells. *n* = 3. (*B*) Immunoblot analyses of control transfected GFP-lamin A (LA) and GFP-progerin fibroblasts as well as GFP-progerin fibroblasts transfected with myc-LAP2α in total and soluble ECM (DOC) fractions using anti-Col12A1, anti-Col1A1, and anti-Timp2 antibodies, with Col4A1 as a loading control for soluble fraction and γ-tubulin as a control for total cell lysate (*C*) GFP-progerin-expressing cells were plated in the absence of ECM or in the presence of ECM derived from GFP-progerin-expressing, wild-type (WT), or GFP-progerin/LAP2α-expressing cells, and proliferation was monitored every other day for 8 d starting from day 4 of induction. *n* = 3. (*D*) LAP2α and lamin A/C chromatin immunoprecipitation (ChIP)-qPCR analysis for deregulated ECM genes *Timp2* and *Col12A1*. *n* = 2.

Next, we tested the effect of myc-LAP2α expression on ECM expression. Intriguingly, in GFP-progerin cells, expression of myc-LAP2α increased the mRNA levels of several ECM proteins twofold to ninefold, while, in GFP-lamin A-expressing cells, myc-LAP2α expression further decreased ECM levels (Fig. 5A). In addition, immunoblots of total and soluble ECM fractions revealed down-regulation of at least three ECM proteins (*Col12A1*, *Col1A1*, and *Timp2*) in progerin-expressing cells, and re-expression of LAP2α increased protein levels (Fig. 5B).

Cultivation of GFP-progerin-expressing cells on preformed ECM derived from either wild-type, progerin-expressing, or progerin/LAP2α-expressing cells for 8 d promoted proliferation compared with cultures on empty plates (Fig. 5C) Interestingly, the ECM derived from progerin cells re-expressing LAP2α displayed the most prominent proliferation-promoting effect (Fig. 5C). Proliferation of wild-type cells was not significantly improved when grown on wild-type ECM in comparison with growth in the absence of ECM (Supplemental Fig. S5C).

LAP2α associates with chromatin throughout the genome and affects chromatin association of other proteins, including high-mobility group protein N5 (Zhang et al. 2013) and lamin A/C (our unpublished data). To investigate potential mechanisms concerning how LAP2α may affect ECM gene expression, we performed LAP2α and lamin A/C chromatin immunoprecipitation (ChIP) in progerin-expressing and lamin A-expressing hTERT cells and tested the association of LAP2α and lamin A/C with ECM genes *Timp2* and *Col12A1* (down-regulated in progerin-expressing cells) by qPCR. LAP2α and lamin A/C bound to both ECM genes in all cells, but, interestingly, gene-associated lamin A/C levels were higher in progerin-expressing versus lamin A-expressing and control cells (Fig. 5D). As lamin A binding to genes was linked to gene repression (Kind et al. 2013; Lund et al. 2013), gain of lamin A association in genomic regions containing ECM genes in progeria cells may contribute to gene repression.

Overall, our results show that progerin expression causes down-regulation of LAP2α at the transcriptional level and that loss of LAP2α contributes to impaired cell proliferation. Furthermore, introduction of LAP2α in the progerin-expressing cells rescues the proliferative defect presumably by increasing ECM expression independently of nucleoplasmic lamins A/C.

## Discussion

In this study, we analyzed the kinetics and consequences of the previously reported decrease in LAP2α levels in HGPS cells (Scaffidi and Misteli 2005, 2008; Cenni et al. 2011; Zhang et al. 2011) and investigated whether LAP2α down-regulation may be causally involved in proliferation defects of HGPS cells (Burtner and Kennedy 2010; Ghosh and Zhou 2014; Gordon et al. 2014). In all HGPS cell cultures analyzed, LAP2α showed a highly heterogeneous distribution of expression, ranging from cells with high/normal LAP2α levels to cells with low or undetectable LAP2α expression, which was inversely correlated with progerin expression levels. In patient cells, LAP2α levels decreased progressively with age of patients and passage number in culture, while progerin levels increased, consistent with previous observations (McClintock et al. 2006; Cao et al. 2011).

**Figure 6.**
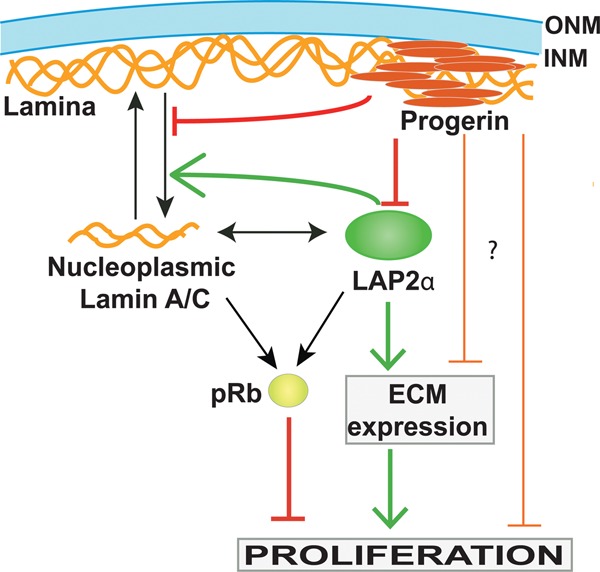
Model depicting the potential role of LAP2α in cell proliferation. Lamin A/C is found at the peripheral lamina and the nucleoplasm, where it interacts with LAP2α and pRb, contributing to proliferation arrest. In addition, LAP2α increases expression of ECM proteins in a lamin A/C-independent manner, which in turn promotes proliferation. Progerin expression causes loss of nucleoplasmic A-type lamins and down-regulation of LAP2α, which in turn impairs proliferation. LAP2α expression in progerin-expressing cells does not rescue nucleoplasmic lamins but rescues ECM expression and cell proliferation.

Our data in patient fibroblasts clearly revealed LAP2α down-regulation at the mRNA and protein levels and showed an inverse correlation with progerin expression. However, the heterogeneity in the cultures precluded further analyses of potential mechanisms involved and did not allow addressing the question of whether LAP2α down-regulation is a consequence or cause of the HGPS-linked proliferation phenotype. To overcome this problem, we generated a tightly controllable cell system in which hTERT immortalized wild-type skin fibroblasts were engineered to allow doxycycline-inducible expression of GFP-progerin or GFP-lamin A as a control. In these cells, we confirmed that LAP2α was progressively down-regulated upon GFP-progerin expression. Since previous studies have revealed that LAP2α is down-regulated in noncycling wild-type cells, including quiescent, senescent, or differentiated cells (Markiewicz et al. 2002, 2005; Naetar et al. 2007), the observed down-regulation of LAP2α in progerin-expressing cells may be a consequence of progerin-mediated cell cycle exit. However, LAP2α mRNA down-regulation in these cells occurred already at a time point when the cells had no detectable proliferation defects. This finding provides strong evidence that LAP2α down-regulation is not a consequence of progerin-linked cell cycle exit but contributes actively to cell proliferation impairment (Fig. 6). This hypothesis was further supported by our result that ectopic expression of LAP2α in progerin-expressing cells rescued cell proliferation.

The mechanism for how progerin expression may cause a decrease in LAP2α mRNA levels is not completely clear. One possibility is that LAP2α down-regulation is caused by an impaired pRb pathway reported in HGPS cells (Dechat et al. 2007; Marji et al. 2010), as LAP2α is a direct target of E2F/pRb-mediated transcriptional regulation (Parise et al. 2006) and was found up-regulated in several cancer types associated with pRb defects (for review, see Brachner and Foisner 2014). In line with this, we found E2F-1 protein levels and some of its target genes down-regulated in progerin-expressing cells. As this correlated with loss of LAP2α, decreased E2F activity in progeria cells may account for the observed decrease in LAP2α mRNA levels. In addition, we found a shift from the hyperphosphorylated to the hypophosphorylated form of pRb, which serves as a transcriptional repressor of pRb target genes (Weinberg 1995) in HGPS patient cells (data not shown). Progerin-mediated changes in gene expression (including that of LAP2α) could also be caused by alterations in higher-order chromatin organization and epigenetic pathways previously reported in progeria cells (Shumaker et al. 2006; McCord et al. 2013).

What are the downstream effects of LAP2α down-regulation in progerin-expressing cells? LAP2α is a direct binding partner of A-type lamins in the nuclear interior (Dechat et al. 2000) and is required to maintain the nucleoplasmic pool of A-type lamins (Fig. 6; Naetar et al. 2008). In line with this mechanism, we found loss of nucleoplasmic A-type lamins in progerin-expressing but not lamin A-expressing fibroblasts. However, reintroduction of ectopic LAP2α in progerin-expressing cells did not rescue the nucleoplasmic localization of A-type lamins, indicating that additional LAP2α-independent mechanisms regulate the localization of A-type lamins in progerin-expressing cells. Since progerin, unlike mature lamin A, is permanently farnesylated at its C terminus and thus is tightly associated with the nuclear membrane (Cao et al. 2007; Dechat et al. 2007), the formation of progerin–lamin A hetero-oligomers at the nuclear membrane may impair efficient LAP2α-mediated translocation of A-type lamins from the lamina to the nuclear interior (Dahl et al. 2006; Delbarre et al. 2006).

In line with previous studies (Bridger and Kill 2004; McClintock et al. 2006), we detected a progressive impairment of cell proliferation and cell cycle exit with increasing expression of progerin in both hTERT fibroblasts and patient cells. Furthermore, our findings that LAP2α mRNA is down-regulated prior to detectable cell proliferation defects and that ectopic expression of LAP2α rescued cell proliferation suggests that LAP2α loss is causally involved in cell cycle exit in progerin-expressing cells. This observation seems puzzling and in disagreement with our previous results showing that loss of LAP2α in mouse cells increased proliferation (Naetar et al. 2008), while its overexpression caused cell cycle exit (Dorner et al. 2006). In line with these previous findings, ectopic expression of LAP2α in the GFP-lamin A-expressing cells slowed down cell proliferation.

Why, then, does expression of LAP2α in progerin-expressing cells increase proliferation? One possible explanation for this conundrum is the observed loss of nucleoplasmic A-type lamins exclusively in progerin-expressing cells. Our previous studies clearly indicate that both LAP2α and nucleoplasmic A-type lamins are required to mediate cell cycle arrest, most likely in a pRb-dependent manner (Dorner et al. 2006; Naetar et al. 2008; Pilat et al. 2013).

These data led to the hypothesis that ectopic LAP2α expression in progerin-expressing cells rescues cell proliferation through an A-type lamin-independent function (Fig. 6). In the search for a potential nucleoplasmic lamin A/C-independent mechanism, we revisited previously reported phenotypes in LAP2α-deficient cells. A gene expression profiling study in LAP2α-deficient versus wild-type mouse myoblasts revealed a prominent down-regulation of several ECM genes (Gotic et al. 2010). Down-regulation of ECM components was also previously observed in HGPS cells and mouse fibroblasts derived from a progeria mouse model (Hernandez et al. 2010), and culturing progerin or prelamin A-expressing cells on an ECM derived from wild-type cells rescued proliferation defects (Hernandez et al. 2010; de la Rosa et al. 2013). In this study, we also found reduced ECM expression in progerin-expressing cells, and ectopic expression of LAP2α rescued ECM expression and proliferation. Furthermore, cultivation of progerin-expressing cells on a preformed ECM derived from GFP-progerin cells re-expressing LAP2α promoted their proliferation as compared with growth on either GFP-progerin-derived or wild-type-derived matrix. Thus, LAP2α down-regulation in progerin-expressing cells may cause defective ECM gene expression, which in turn impairs cell proliferation.

How can LAP2α affect expression of ECM genes? LAP2α is a member of the chromatin-binding LEM protein family (Brachner and Foisner 2011) and harbors several chromatin-binding motifs: a LEM domain mediating the interaction with the DNA-binding protein barrier to autointegration factor (BAF), a LEM-like motive mediating interaction with DNA directly (Cai et al. 2001), and a chromatin targeting domain involved in chromatin binding of LAP2α during post-mitotic nuclear assembly (Vlcek et al. 1999). More recent studies showed that LAP2α interacts with long regions in chromatin throughout the genome, presumably in a DNA sequence-independent manner, and affects chromatin interaction of the high-mobility group protein N5 (Zhang et al. 2013), which in turn likely impinges on chromatin organization and compaction, resulting in gene expression changes. Furthermore, our unpublished data indicate that LAP2α may affect lamin A/C's chromatin binding. In line with this hypothesis, we found that LAP2α and lamin A/C associate with ECM genes in control, lamin A-expressing, and progerin-expressing cells, but lamin A/C association with down-regulated ECM genes was increased in progerin-expressing versus control cells. In view of previous data linking lamin A/C binding in genomic regions at and around genes to gene repression (Kind et al. 2013; Lund et al. 2013), the increased lamin A/C association with ECM genes may contribute to their repression. Thus, our data are consistent with the hypothesis that LAP2α may contribute to chromatin organization and gene expression.

Overall, our study reveals a novel function of LAP2α in regulating ECM protein expression that is independent of nucleoplasmic LAP2α–lamin A/C complexes. Loss of LAP2α in progeria cells and a concomitant impairment of ECM production may thus causally contribute to the proliferation defect observed in HGPS cells.

## Materials and methods

### Cell culture

hTERT-Teton-Pro cell lines were generated by serial infection of previously described hTERT immortalized human dermal fibroblasts (Scaffidi and Misteli 2008) with lentivirus generated from both the pLenti CMV rtTA3 Hygro plasmid (Addgene) encoding for the tetracycline-controlled transactivator 3 and pLenti CMV TRE3G Neo vectors encoding for either GFP-lamin A or GFP-progerin. The latter were created by BamHI–EcoRI-mediated ligation of GFP-lamin A and GFP-progerin from the pBabe puro GFP-progerin and pBabe puro GFP-lamin A plasmids (Scaffidi and Misteli 2008) into the pENTR1A no ccDB vector (Addgene) followed by Gateway-mediated LR recombination (Invitrogen), according to manufacturer's instructions, into the pLenti CMV TRE3G Neo plasmid (Addgene). After antibiotic selection (200 μg/mL G418 and 200 μg/mL hygromycin), individual clones were selected for GFP-lamin A and GFP-progerin levels equal to endogenous lamin A expression levels after 4 d of induction with 1 μg/mL doxycycline. hTERT-TetOn-Pro cell lines were maintained in Dulbeco's modified minimum essential medium (DMEM) (Invitrogen/GIBCO) containing 15% Tet-free fetal bovine serum (FBS), 2 mM L-glutamine, 100 U/mL penicillin, and 100 μg/mL streptomycin, with the addition of G418 and hygromycin as described above. Protein expression was induced by adding 1 μg/mL doxycycline for the indicated time points.

Primary dermal fibroblast cell lines from patients and healthy donors were obtained from the Coriell Cell Repository (CCR) and the Progeria Research Foundation (PRF). The HGPS cell lines used were AG11513 (12 yr, CCR, 10 population doublings [PDs]), HGADFN155 (1 yr, PRF, 10 PDs), HGADFN167 (8 yr, PRF, 10 PDs), and HGADFN003 (2 yr, PRF, 10 PDs). The control cell lines used were GM04390 (23 yr, CCR, 7.3 PDs), GM02037 (13 yr, CCR), HGMDFN168 (40 yr, PRF, 10 PDs), and HGMDFN090 (37 yr, PRF, 10 PDs). For consistency, throughout this study, cell line HGADFN003 was named HGPS 1, HGADFN155 was named HGPS 2, AG11513 was named HGPS 3, HGADFN168 was named WT 1, and GM02037 was named WT 2. Primary fibroblasts were cultured in DMEM supplemented with 15% FBS, 2 mM L-glutamine, 100 U/mL penicillin, and 100 μg/mL streptomycin at 37°C in 5% CO_2_.

### Plasmids, cloning, and retroviral transfections

The retroviral plasmid pHR′ CMV mCherry (modified from Adgene pHR′ CMV GFP plasmid #14858) was kindly provided by I. Yudushkin (Max F. Perutz Laboratories, Vienna, Austria), and the control pHR′ CMV GFP retroviral plasmid (Adgene, plasmid #14858) was provided by D. Blaas (Max F. Perutz Laboratories, Vienna, Austria). For generation of human myc-LAP2α-pHR, retroviral construct pHR′ CMV mCherry plasmid was modified as follows: The mCherry cassette was deleted with NotI and BamHI restriction enzymes, and the SpeI restriction site was introduced by insertion of oligonucleotides 5′-GATCCCGACTAGTCGGC-3′ and 5′-GGCCGCCGACTAGTCGG-3′, creating pHR′ CMV SpeI. cDNA encoding human myc-LAP2α was amplified from pTD15 (Vlcek et al. 1999) by PCR using 5′-GGCACTAGTATGCCGGAGTTCCTGGAAGACCCCTCG-3′ and 5′-GGCACTAGTCTAGACATTCAAGTCCTCTTCAGCCCTG-3′ primers and cloned into pHR′ CMV SpeI using Spe1, generating human myc-LAP2α-pHR plasmid.

For retroviral production, 50% confluent HEK293T cells were cotransfected with psPAX2 packaging plasmid and pMD2.G envelope plasmid (Adgene; provided by I. Yudushkin) together with the myc-LAP2α-pHR or GFP-expressing retroviral vector using polyethylenimine (PEI) (Polysciences) and maintained in 10 mL of DMEM supplemented with 10% FBS, 2 mM L-glutamine, and antibiotics in 10-cm plates. Viral supernatants were collected at 48 h, 72 h, and 96 h after transfection. The 48-h supernatant was filtered through a 0.45-µm filter and concentrated using Retro-X concentrator (Clontech) according to the manufacturer's instructions. The 72-h supernatant was filtered and mixed with the concentrated 48-h supernatant and added to GFP-lamin A or GFP-progerin hTERT-TetOn-Pro cells or primary patient cells seeded at a density of 10^5^ cells per well in a six-well plate. To increase infection efficiency, cells were spun at 1000*g* for 90 min at room temperature immediately after addition of the retroviral supernatant. After 24 h of incubation at 37°C, the viral supernatant was replaced with the 96-h supernatant and incubated for another 24 h.

### Cell-derived matrix preparation

Eight days after plating, GFP-lamin A-expressing cells were treated with 20 mM NH_4_OH for 5 min at room temperature and washed gently with PBS (+Ca^2+^, +Mg^2+^), followed by a 30-min incubation with 10 μg/mL DNase I in PBS (+Ca^2+^, +Mg^2+^) at 37°C. Treated plates were gently washed three times with dH_2_O, and the cell-derived ECM was immediately used for cultivation of the cells (modified from Cseh et al. 2010). For immunoblot analysis, cells were grown on 6-cm plates until they reached confluency and left for three additional days to produce matrix. Cells were washed with ice-cold PBS and extracted in ice-cold DOC lysis buffer (2 mM EDTA, 20 mM Tris at pH 8.8, 1mM DTT, 2% sodium deoxycholate, DOC) for 15 min on ice. Lysates were collected and sonicated for 10 sec at 45% power output (total DOC fraction). Soluble and insoluble DOC fractions were obtained by centrifugation at 14,000 rpm for 45 min at 4°C (Eppendorf, 5417R). Protein concentration was determined using a Pierce BCA protein assay kit (Thermo Scientific).

### Antibodies

Primary antibodies used for immunofluorescence and immunoblotting were rabbit antiserum to LAP2α (245.2) (Vlcek et al. 2002), mouse monoclonal anti-LAP2α (15/2) (Vlcek et al. 2002), mouse monoclonal antibody to LAP2α (hybridoma supernatant) (Alexis Biochemicals, 8C10-1H11), goat polyclonal anti-Lamin A/C (Santa Cruz Biotechnology, N-18), mouse monoclonal anti-Lamin A/C (clone 4C11, provided by E. Ogris, Max F. Perutz Laboratories, Vienna, Austria) (Roblek et al. 2010), rabbit polyclonal anti-lamin A (Dechat et al. 2007), and rabbit polyclonal anti-lamin C (kindly provided by R. Goldman, Northwestern University, Chicago, IL) (Kochin et al. 2014), mouse monoclonal anti-progerin (clone 13A4, provided by E. Ogris) (Alexis Biochemicals), monoclonal mouse anti-myc (provided by E. Ogris) (Alexis Biochemicals), rabbit polyclonal anti-ubiquitin (Cell Signaling, #3933), mouse monoclonal anti-Col12A1 (Santa Cruz Biotechnology, A-11), goat polyclonal anti-Col1A1 (Santa Cruz Biotechnology, D-13), rabbit monoclonal anti-TIMP2 (Cell Signaling), goat polyclonal anti-actin (Santa Cruz Biotechnology, I-19), and mouse monoclonal anti-γ-tubulin (Sigma, GTU-88).

### Immunofluorescence microscopy and image analysis

Cells were grown on glass coverslips, washed with PBS, and fixed with 4% paraformaldehyde for 15 min at room temperature. Cells were washed twice with PBS and treated with 0.5% Triton X-100 and 50 mM NH_4_Cl for 5 min in PBS. Fixed cells were rinsed twice with PBS and blocked for 30 min in blocking buffer (0.2% gelatine in PBS). Primary antibodies were diluted in blocking buffer supplemented with 5% normal goat serum or in blocking buffer only (for the primary goat antibodies), and incubation was performed for 2 h at room temperature. Cells were washed once with 0.05% Tween/PBS and twice with PBS and probed with fluorescently labeled secondary antibodies (DyLight fluor secondary antibodies, Thermo Scientific) diluted in PBS for 1 h at room temperature. All samples were counterstained with DAPI (1:10000 in PBS) for 15 min at room temperature and mounted in glycerol mounting medium (DABCO).

Images were acquired on a confocal microscope (LSM 710, Carl Zeiss) using 40×/1.3 NA oil immersion objective plan-apochromat, or 63×/1.4 NA oil differential interference contrast plan-apochromat (Carl Zeiss). Images were processed and exported using Zen software (Carl Zeiss). Tile scan images used for mean fluorescence intensity measurements were acquired on a 710 confocal microscope (Carl Zeiss) using 25×/0.8 NA oil immersion objective, 1× zoom, and 5 × 5 tile scan. Mean fluorescence intensities were quantified using ImageJ. The intensity of the nucleoplasm and rim was measured using the profile tool in the Zen image software (Carl Zeiss). Peripheral and nucleoplasmic intensity values were determined along a nuclear axis and plotted. Images were processed using Photoshop CS4 (Adobe), and figures were assembled using Illustrator CS3 (Adobe).

### Immunoblotting

Total cell lysates were prepared by dissolving cells of one 6-cm dish in 200 µL of RIPA buffer (50 mM Tris-HCl at pH 8.0, 150 mM NaCl, 1% TX-100, 0.1% SDS, 5 mM EDTA, 1 mM EGTA, 1× Complete protease inhibitor mix [Roche], 1 mM PMSF, 1 mM ortho-sodium vanadate). Lysates were quantified using Pierce BCA protein assay kit (Microplate, Thermo Scientific), separated by SDS-PAGE, and transferred onto PVDF membrane. Membranes were blocked for 30 min in blocking buffer (5% BSA/PBST, 0.05% Tween 20) and incubated with primary antibody diluted in 2% BSA/PBST and 0.05% Tween 20 overnight at 4°C. Membranes were washed three times for 15 min in PBS and 0.05% Tween 20 and incubated with fluorescently labeled secondary antibodies (IRDye, Licor) for 1 h at room temperature. Quantification of protein levels was performed with Licor Odyssey infrared imaging system and normalized to actin or γ-tubulin.

### Quantitative real-time PCR

RNA was isolated using the RNeasy minikit (Qiagen), and total RNA was quantified by a spectrophotometer (NanoDrop Technologies, ND-1000). cDNA was generated using RevertAid reverse transcriptase (Thermo Scientific). qRT–PCR was performed using the primers listed in Supplemental Table 1. All reactions were carried out in triplicates on an Eppendorf Realplex 2 Mastercycler with KAPA SYBR Green PCR master mix (Peqlab) according to the manufacturer's instructions. To normalize for mRNA input, relative expression levels were calculated by normalizing to endogenous β-actin and HPRT levels. For consistency, all results are shown relative to β-actin levels. Expression values are shown as means ± SD of biological triplicates.

### Growth curves and proliferation assays

hTERT-TetOn-Pro cell lines were plated in triplicates on six-well plates at a density of 40,000 cells per well and grown for 10 d under various conditions (uninduced or induced with doxycycline with or without h-myc-LAP2α). Cell numbers were determined at the indicated time points using a CASY counter system. EdU incorporation assays were performed using EdU-Click imaging kit according to the manufacturer's instructions (Base Click).

### ChIP

ChIP was performed as previously described (Hauser et al. 2002) with few modifications: Asynchronously growing cells were washed in PBS^++^ (supplemented with 1 mM Ca^2+^, 0.5 mM Mg^2+^) and cross-linked in 1% formaldehyde and PBS^++^ for 10 min at room temperature. Cross-linking was stopped by the addition of glycine to a final concentration of 125 mM. Cells were washed and scraped into ice-cold PBS^++^ containing 1 mM PMSF and 1× Complete protease inhibitor mix (Roche) and collected by centrifugation at 2000*g* for 5 min at 4°C. Cell pellets were resuspended in wash buffer I (10 mM HEPES at pH 7.5, 0.25% Triton X-100, 10 mM EDTA, 0.5 mM EGTA, 1 mM PMSF, 1× Complete protease inhibitor mix), incubated for 10 min on ice, and centrifuged at 2000*g* for 5 min at 4°C. Washing was repeated with wash buffer II (10 mM HEPES at pH 7.5, 0.2 M NaCl, 1 mM EDTA, 0.5 mM EGTA), and pellets were resuspended in lysis buffer (50 mM Tris-HCl at pH 8.1, 10 mM EDTA, 1% SDS) and sonicated with a Bioruptor Plus (Diagenode) for 20 cycles (30 sec on/off on the “high” setting at 4°C). The sheared chromatin was cleared twice by centrifugation at 14.000*g* for 10 min at 8°C (input). Input chromatin (50 µg) was diluted 1:10 in ChIP dilution buffer (16.7 mM Tris-HCl at pH 8.1, 167 mM NaCl, 1.2 mM EDTA, 1.1 % Triton X-100, 0.01% SDS), and 10 µg of anti-lamin A/C (Santa Cruz Biotechnology, N-18) and 10 µL of rabbit antiserum to LAP2α (245.2) were added and incubated overnight at 4°C. Chromatin–antibody complexes were collected with 30 µL of magnetic protein A/G beads (Pierce) for 4–5 h at 4°C and washed once each with RIPA buffer (50 mM Tris-HCl at pH 8.0, 150 mM NaCl, 0.1% SDS, 0.5% sodium deoxycholate, 1% NP-40), high-salt buffer (50 mM Tris at pH 8.0, 500 mM NaCl, 0.1% SDS, 1% NP-40), and LiCl buffer (50 mM Tris at pH 8.0, 25 mM LiCl, 0.5% sodium deoxycholate, 1% NP-40) and twice with TE buffer (10 mM Tris at pH 8.0, 1 mM EDTA). Chromatin antibody complexes were eluted from beads in 200 µL of 2% SDS, 0.1 M NaHCO_3_, and 10 mM dithiothreitol, and cross-links were reversed by addition of 0.05 vol of 4 M NaCl and overnight incubation at 65°C. After addition of 0.025 vol of 0.5 M EDTA and 0.05 vol of 1 M Tris-HCl (pH 6.5), digestion was performed with 4 µg of proteinase K (Ambion, catalog no. AM2546) for 1 h at 55°C. Eluted DNA was purified with the ChIP DNA Clean and Concentrator kit (Zymoresearch, catalog no. D5205) according to the manufacturer's protocol.

### Statistical analysis

A two-tailed Student's *t*-test was used for statistical analyses. The calculations were done in Microsoft Excel or GraphPad Prism software. All experimental data are reported as the mean of three biological replicates, except for the growth curves, where each experiment was done in triplicates, and the means of at least three different experiments were calculated for each time point. Error bars represent SD, except for the results of the growth curves, where the error bars represent SEM. Statistical significance was classified as follows: *P* < 0.05 (*), *P* < 0.005 (**), and *P* < 0.0005 (***).

## Supplementary Material

Supplemental Material
