# Supplementary material for: Proliferation of progeria cells is enhanced by lamina-associated polypeptide 2α (LAP2α) through expression of extracellular matrix proteins
Source: Genes Dev. 2015 Oct 1;29(19):2022–36. doi: 10.1101/gad.263939.115 (PMC4604344; doi:10.1101/gad.263939.115)
Supplement: Supplemental Material [file supp_29_19_2022__index.html]

Supplemental Material 

# Proliferation of progeria cells is enhanced by lamina-associated polypeptide 2α (LAP2α) through expression of extracellular matrix proteins

## Supplemental Material

**Files in this Data Supplement:**

- Supp Material.pdf
