# Supplementary material for: Proliferation of progeria cells is enhanced by lamina-associated polypeptide 2α (LAP2α) through expression of extracellular matrix proteins
Source: Genes Dev. 2015 Oct 1;29(19):2022–36. doi: 10.1101/gad.263939.115 (PMC4604344; doi:10.1101/gad.263939.115)
Supplement: Supplemental Material [file supp_29.19.2022_SuppMaterial.pdf]

Supplemental material, Vidak et al.

Table S1. (List of primers used for quantitative real-time PCR analysis)

Figure S1. (related to Figure 1)

Figure S2. (Characterization of the generated human TERT-immortalized skin fibroblast cell lines containing doxycycline-inducible GFP-lamin A or GFP–progerin)

Figure S3. (Potential mechanisms of LAPα downregulation upon progerin expression, related to Figure 3)

Figure S4. (related to Figure 4)

Figure S5. (related to Figure 5)

Table S1. Primers used for quantitative real-time PCR analysis.

Primer	Sequence
**LAP2α F**	TCCTTTGGGCAGTACCGAAC
**LAP2α R**	AGACCAACATGGCACTGTG
**Col12A1 F**	GCCACTGAAGAAGTTAGAGGGA
**Col12A1 R**	TTTCACTTTTCCTGGTGCCC
**Col11A1 F**	ACCTGACCTGCCGTCTAGAA
**Col11A1 R**	TCCACCACCCTGTTGCTGTA
**Col1A1 F**	CATGACCGAGACGTGTGTGGAAACC
**Col1A1 R**	CATGACCGAGACGTGTGTGGAAACC
**Cox 1 F**	AGCATCTACGGTTTGCTGTG
**Cox 1 R**	TACTCTGTTGTGTTCCCGCA
**Aspn F**	TTTGAAGGGGTGACGGTGTT
**Aspn R**	AGTTGGTGGTAAGCCTTTAGGA
**Timp2 F**	GGCAAGATGCACATCACCCTCTGT
**Timp2 R**	GTTCTTCTCTGTGACCCAGTCCATCC
**Col3A1 F**	GGATCAGGCCAGTGGAAATGTAAAGA
**Col3A1 R**	CTTGCGTGTTCGATATTCAAAGACTGTT
**MMP15 F**	CGACTGGGGCAGGGTGTTTAGA
**MMP15 R**	GACAGTCTCCAACTGGGCAAAGAGAG
**TK 2 F**	AATCAGTGATCTGTGTCGAGGG
**TK 2 R**	TTGTGGCCACGGACATTTCT
**PCNA F**	TGTCGATAAAGAGGAGGAAGC
**PCNA R**	AAGAGAGTGGAGTGGCTTTTG
**CENPF F**	ACAGCTGGTGGCAGCAGATCAC
**CENPF R**	GGGACAAGTCGGCCTCGCTTG
**AurA F**	TGGCAAATGCCCTGTCTTACTGTCA
**AurA R**	GGGGGCAGGTAGTCCAGGGT
**β-actin F**	ATAGCACAGCCTGGATAGCAACGTAC
**β-actin R**	CACCTTCTACAATGAGCTGCGTGTG
**HPRT F**	TCAGGCAGTATAATCCAAAGATGGT
**HPRT R**	AGTCTGGCTTATATCCAACACTTCG
**COL12A1 (ex5/ex6) F**	ACGGTTCTGTGGAAAACCAA
**COL12A1 (ex5/ex6) R**	CGCACTGAAAAGGACAGTGA
**TIMP2 (ex5) F**	AGTGTCCTGGAGGCTGAGAA
**TIMP2 (ex5) R**	GAAAAAGCTGGGTCTTGCTG

Supplemental Figure Legends

Figure S1. LAP2α levels and the nucleoplasmic pool of lamin A are decreased in HGPS fibroblasts. (A) HGPS and wild-type (WT) fibroblasts were analyzed by immunofluorescence microscopy using anti-LAP2α antibody (green) and DAPI (DNA, blue). Scale bar: 20μm. (B) Mean fluorescence intensities of LAP2α (top) and progerin (bottom) signals were measured in 250 nuclei of 1 WT and 3 different HGPS cell lines (HGPS 1-HGADFN003, p13; HGPS 2-HGADFN155, p12 and HGPS 3-AG11513B, p11). The percentage of LAP2α signal is shown relative to WT. (C) Mean fluorescence intensities of LAP2α (left) and progerin (right) signals in HGPS 2 cell line at passage 10 and 20 (n=3). (D) HGPS or WT fibroblasts were processed for immunofluorescence microscopy using anti-lamin A (LA, green, does not react with LC or progerin) and anti-progerin (red) antibodies. DNA was stained with DAPI (blue). Bar represents 10 μm. (E) Mean fluorescence intensity of the lamin A signal was measured across nuclei (dotted line) and plotted.

Figure S2. Ectopic expression of GFP-lamin A and GFP-progerin in hTERT-immortalized skin fibroblasts. (A) hTERT-immortalized Tet-on skin fibroblasts allowing doxycycline-inducible expression of GFP-lamin A (LA) or GFP-progerin were analyzed by fluorescence microscopy in their uninduced state (- Dox) and 1 d post induction with doxycycline (+ Dox). DNA was stained with DAPI. Scale bar: 20 μm. (B) Immunoblot analysis of total cell lysates using anti-lamin A/C and anti-actin antibody as a loading control. (C) Average mean GFP fluorescence intensity signal of 250 nuclei prior doxycycline induction and 2 d, 4 d, 6 d and 8 d post induction. (D) hTERT fibroblasts were transfected with empty vector (pLenti CMV rtTA3) and proliferation was monitored in the absence and presence of doxycycline for 10 days.

Figure S3. Potential mechanisms of LAP2α downregulation. (A) Immunoblot analysis of total cell lysates of wild type (WT) and HGPS primary fibroblasts after treatment with DMSO or proteasomal inhibitor MG132 (1μM) for 2 h and 24 h, using anti-LAP2α, anti-actin and anti-ubiquitin antibodies. Note that the ubiquitin signal is shifted to high molecular weight bands in MG132-treated samples, confirming efficient proteasomal inhibition. (B) Immunoblot analysis of LAP2α protein in wild type versus $LMNA^{-/-}$ mouse embryonic fibroblasts. Actin served as loading control. (C) GFP-LA and GFP-progerin hTERT cells (left) and HGPS and wild-type (WT) primary fibroblasts (right) were analyzed by immunofluorescence microscopy using anti-E2F-1 (purple; E2F-1, c-20, Santa Cruz) and anti-LAP2α (red) antibodies. GFP fusion proteins were detected by GFP fluorescence (green), DNA with DAPI (blue). Scale bar: 20μm. Note that E2F-1 is downregulated in cells showing low LAP2α signal. (D) Quantitative RT-PCR expression analysis of E2F target genes in hTERT cells (left panel) and in primary human fibroblasts (right panel) relative to β-actin and normalized to their respective uninduced or WT values (n=3).

Figure S4. Ectopic expression of h-myc-LAP2α in uninduced hTERT-immortalized skin fibroblasts. (A) Immunofluorescence analysis of h-myc-LAP2α expression in uninduced (-Dox) hTERT-immortalized fibroblasts using α-myc antibody (red) and DAPI (blue). Scale bar: 20 μm. (B) Immunoblot analysis of total cell lysates of GFP-expressing control and h-myc-LAP2α expressing cells using anti-myc and anti-LAP2α specific antibodies. Actin levels served as a loading control. (C) Uninduced cells were transfected either with a control GFP-expressing construct (pHR-GFP) or h-myc-LAP2α- expressing construct on two consecutive days, grown in a medium w/o doxycycline (-Dox) and counted every other day for 6 d (n=3). Note that expression of h-myc-LAP2α causes downregulation of cell proliferation in both cell lines without induction of LA or progerin.

Figure S5. ECM expression is downregulated in progerin-expressing fibroblasts. (A) Quantitative RT-PCR expression analysis of ECM components in primary human HGPS fibroblasts relative to β-actin and normalized to WT values (n=3). (B) ECM expression levels relative to β-actin were determined in hTERT-immortalized fibroblasts by quantitative RT-PCR at 4 d post-induction. The expression levels were normalized to their respective uninduced state (n=3). (C) GFP-LA expressing hTERT fibroblasts were plated in the absence or presence of wild type ECM and their proliferation was monitored for 8 days (n=3).

Vidak_Fig S1

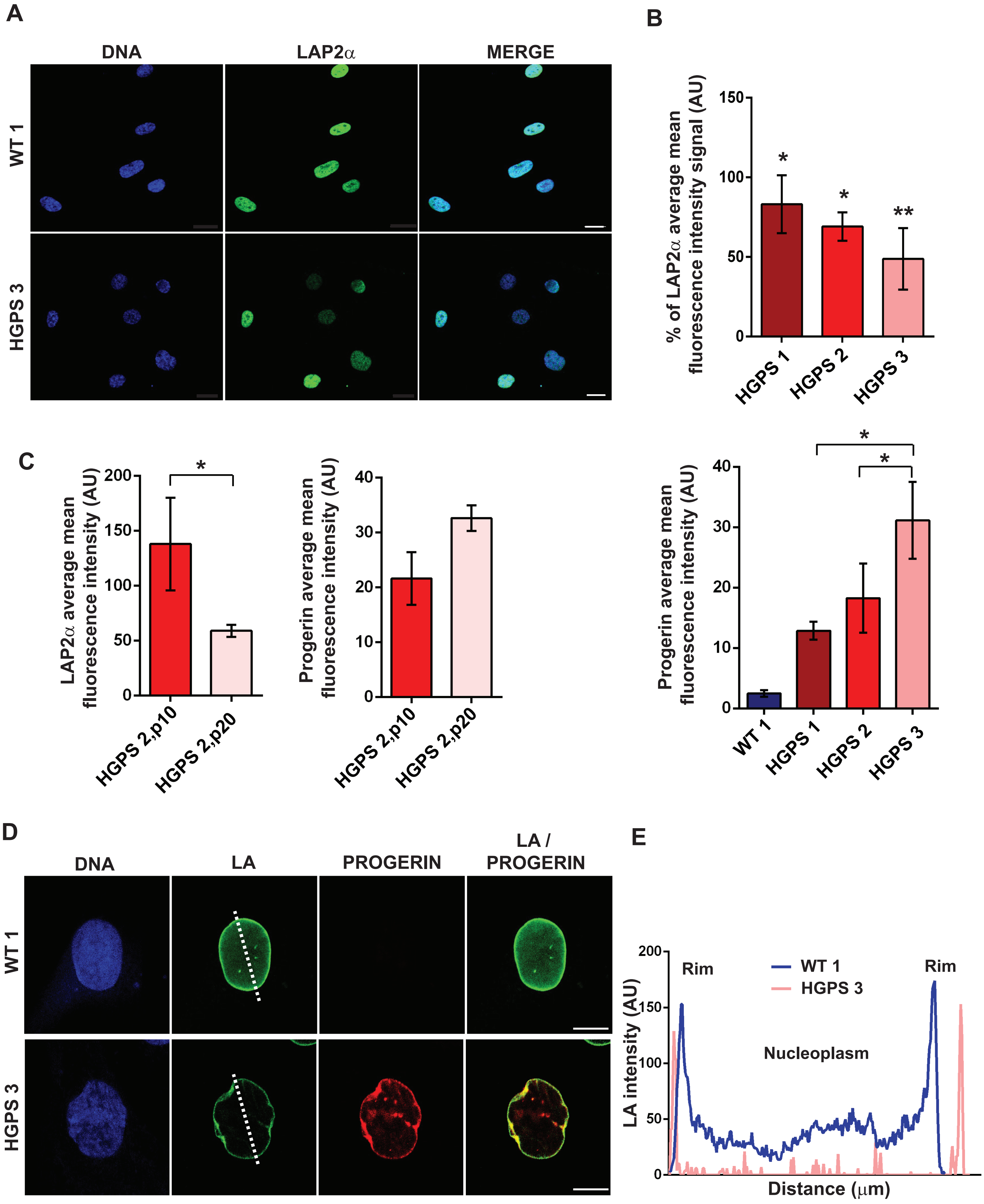
A
DNA
LAP2α
MERGE
WT 1
HGPS 3
B
% of LAP2α average mean fluorescence intensity signal (AU)
150
100
50
0
*
*
**
HGPS 1
HGPS 2
HGPS 3
C
LAP2α average mean fluorescence intensity (AU)
200
150
100
50
0
*
HGPS 2,p10
HGPS 2,p20
Progerin average mean fluorescence intensity (AU)
40
30
20
10
0
HGPS 2,p10
HGPS 2,p20
Progerin average mean fluorescence intensity (AU)
40
30
20
10
0
*
*
WT 1
HGPS 1
HGPS 2
HGPS 3
D
DNA
LA
PROGERIN
LA / PROGERIN
WT 1
HGPS 3
E
LA intensity (AU)
200
150
100
50
0
Rim
Rim
WT 1
HGPS 3
Nucleoplasm
Distance (μm)

Vidak_Fig S2

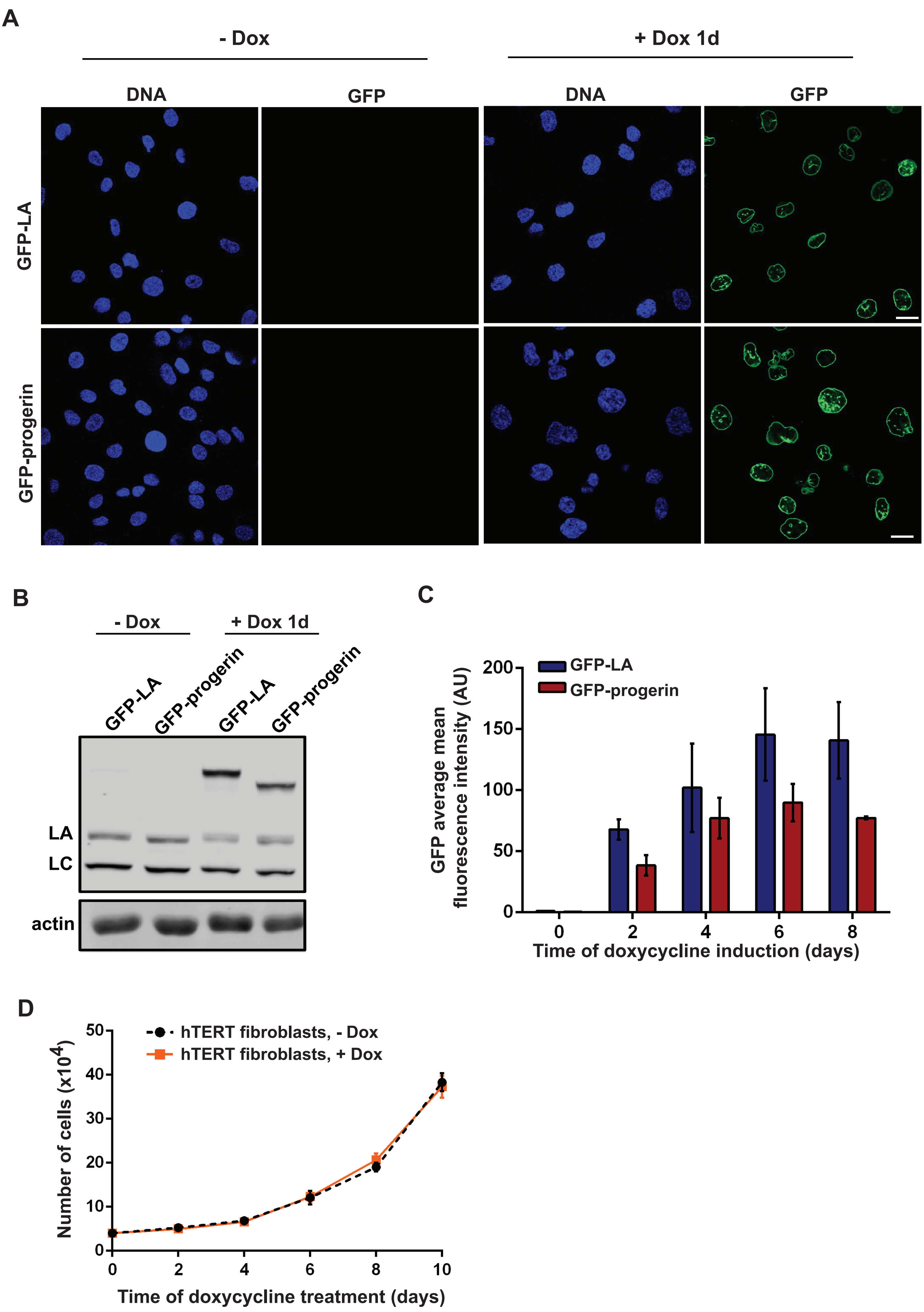

A
- Dox
+ Dox 1d
DNA
GFP
DNA
GFP
GFP-LA
GFP-progerin
B
- Dox
+ Dox 1d
GFP-LA
GFP-progerin
GFP-LA
GFP-progerin
LA
LC
actin
C
GFP-LA
GFP-progerin
GFP average mean fluorescence intensity (AU)
200
150
100
50
0
0
2
4
6
8
Time of doxycycline induction (days)
D
hTERT fibroblasts, - Dox
hTERT fibroblasts, + Dox
Number of cells (x10^4)
50
40
30
20
10
0
0
2
4
6
8
10
Time of doxycycline treatment (days)

Vidak_Fig S3

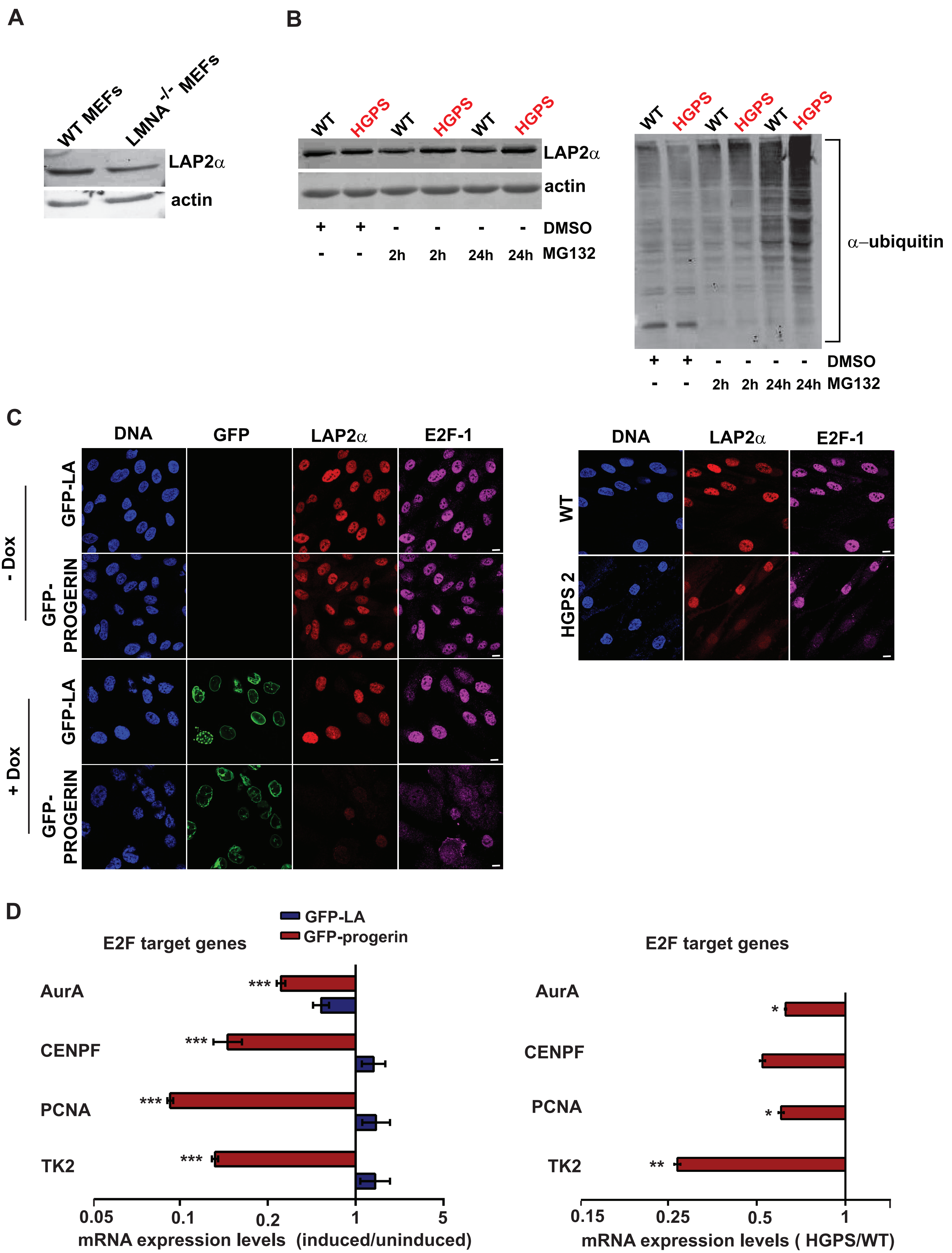

A
WT MEFs
LMNA -/- MEFs
LAP2α
actin
B
WT
HGPS
LAP2α
actin
DMSO
MG132
2h
24h
α–ubiquitin
C
DNA
GFP
LAP2α
E2F-1
GFP-LA
GFP-PROGERIN
- Dox
+ Dox
WT
HGPS 2
D
GFP-LA
GFP-progerin
E2F target genes
AurA
CENPF
PCNA
TK2
0.05
0.1
0.2
1
5
mRNA expression levels (induced/uninduced)
0.15
0.25
0.5
mRNA expression levels (HGPS/WT)

Vidak_Fig S4

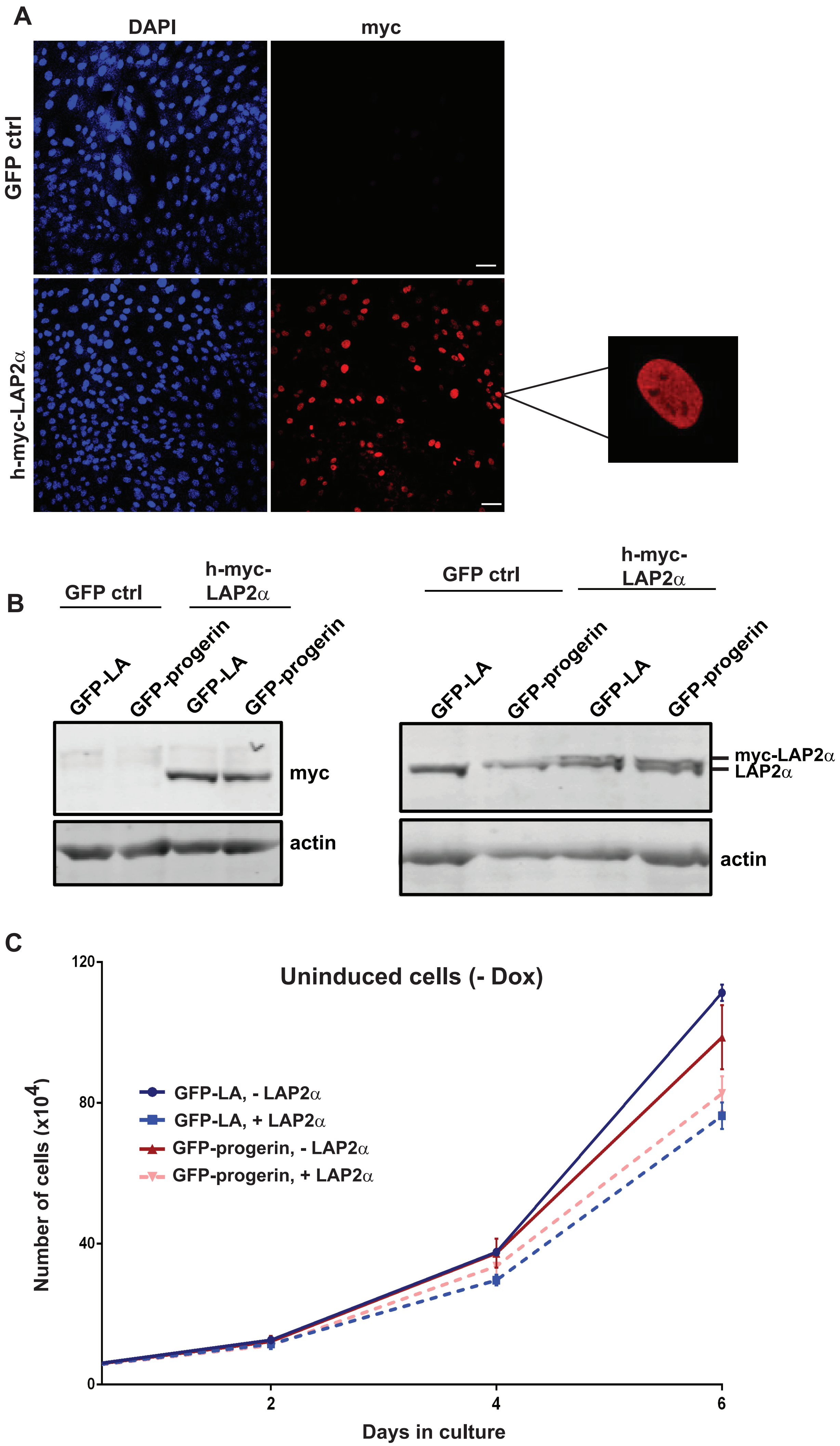
A
DAPI
myc
GFP ctrl
h-myc-LAP2α
B
GFP ctrl
h-myc-LAP2α
GFP-LA
GFP-progerin
GFP-LA
GFP-progerin
myc
actin
GFP ctrl
h-myc-LAP2α
GFP-LA
GFP-progerin
GFP-LA
GFP-progerin
myc-LAP2α
LAP2α
actin
C
Uninduced cells (- Dox)
120
80
40
0
Number of cells (x10^4)
GFP-LA, - LAP2α
GFP-LA, + LAP2α
GFP-progerin, - LAP2α
GFP-progerin, + LAP2α
2
4
6
Days in culture

Vidak_Fig S5

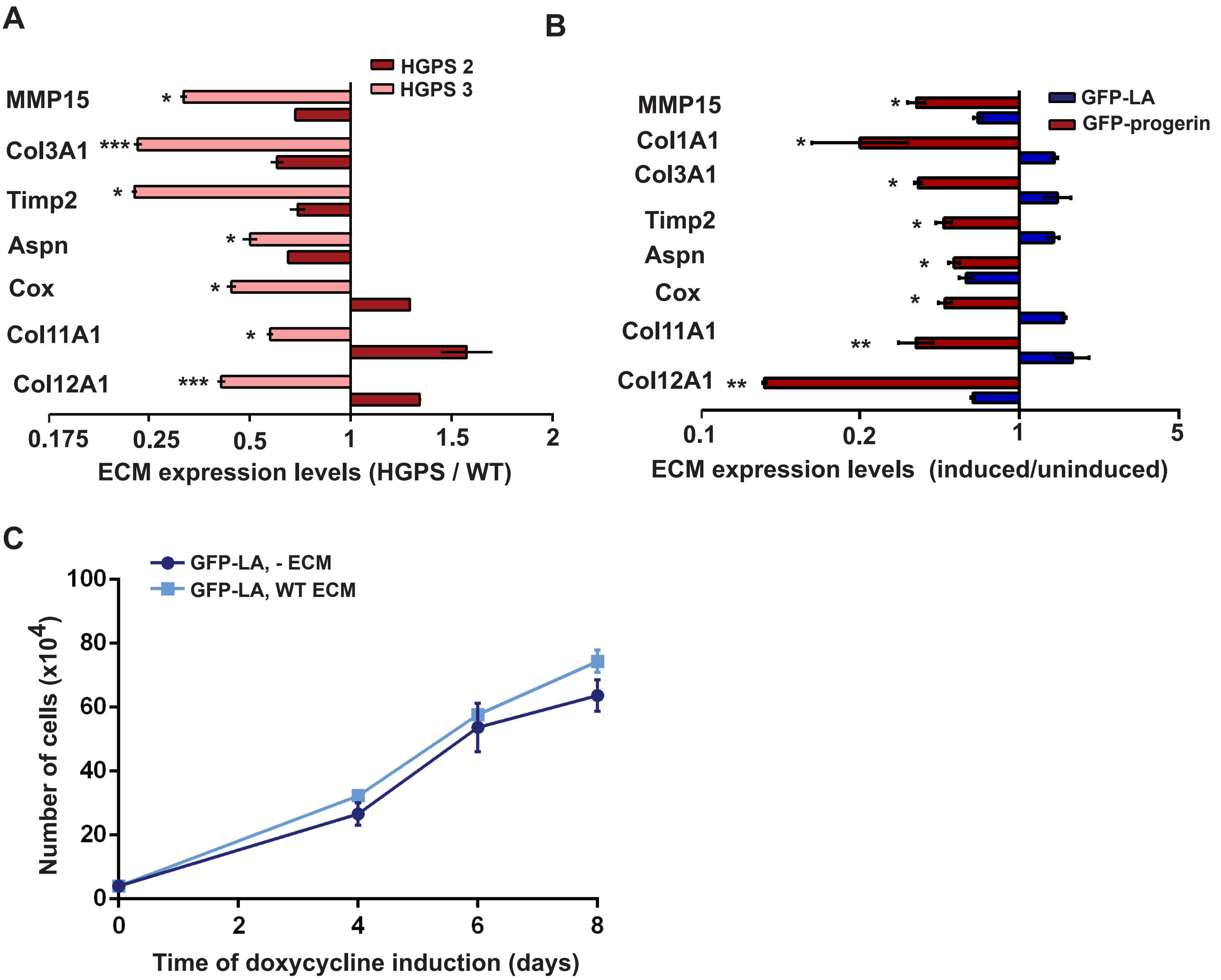
A
HGPS 2
HGPS 3
MMP15
Col3A1
Timp2
Aspn
Cox
Col11A1
Col12A1
*
***
*
*
*
*
***
0.175
0.25
0.5
1
1.5
2
ECM expression levels (HGPS / WT)
B
GFP-LA
GFP-progerin
MMP15
Col1A1
Col3A1
Timp2
Aspn
Cox
Col11A1
Col12A1
*
*
*
*
*
*
**
**
0.1
0.2
1
5
ECM expression levels (induced/uninduced)
C
GFP-LA, - ECM
GFP-LA, WT ECM
Number of cells (x10^4)
100
80
60
40
20
0
0
2
4
6
8
Time of doxycycline induction (days)
